# Predicted Impact of Mass Drug Administration on the Development of Protective Immunity against *Schistosoma haematobium*

**DOI:** 10.1371/journal.pntd.0003059

**Published:** 2014-07-31

**Authors:** Kate M. Mitchell, Francisca Mutapi, Takafira Mduluza, Nicholas Midzi, Nicholas J. Savill, Mark E. J. Woolhouse

**Affiliations:** 1 Centre for Immunity, Infection and Evolution, Institute of Immunology and Infection Research, School of Biological Sciences, University of Edinburgh, Edinburgh, United Kingdom; 2 Department of Biochemistry, University of Zimbabwe, Harare, Zimbabwe; 3 College of Health Sciences, University of KwaZulu Natal, Durban, South Africa; 4 National Institute of Health Research, Harare, Zimbabwe; University of Queensland, Australia

## Abstract

Previous studies suggest that protective immunity against *Schistosoma haematobium* is primarily stimulated by antigens from dying worms. Praziquantel treatment kills adult worms, boosting antigen exposure and protective antibody levels. Current schistosomiasis control efforts use repeated mass drug administration (MDA) of praziquantel to reduce morbidity, and may also reduce transmission. The long-term impact of MDA upon protective immunity, and subsequent effects on infection dynamics, are not known. A stochastic individual-based model describing levels of *S. haematobium* worm burden, egg output and protective parasite-specific antibody, which has previously been fitted to cross-sectional and short-term post-treatment egg count and antibody patterns, was used to predict dynamics of measured egg output and antibody during and after a 5-year MDA campaign. Different treatment schedules based on current World Health Organisation recommendations as well as different assumptions about reductions in transmission were investigated. We found that antibody levels were initially boosted by MDA, but declined below pre-intervention levels during or after MDA if protective immunity was short-lived. Following cessation of MDA, our models predicted that measured egg counts could sometimes overshoot pre-intervention levels, even if MDA had had no effect on transmission. With no reduction in transmission, this overshoot occurred if protective immunity was short-lived. This implies that disease burden may temporarily increase following discontinuation of treatment, even in the absence of any reduction in the overall transmission rate. If MDA was additionally assumed to reduce transmission, a larger overshoot was seen across a wide range of parameter combinations, including those with longer-lived protective immunity. MDA may reduce population levels of immunity to urogenital schistosomiasis in the long-term (3–10 years), particularly if transmission is reduced. If MDA is stopped while *S. haematobium* is still being transmitted, large rebounds (up to a doubling) in egg counts could occur.

## Author Summary

Urogenital schistosomiasis, caused by schistosome blood flukes, infects more than 100 million people in sub-Saharan Africa. Current control efforts involve regularly treating all school-aged children with the drug praziquantel, which kills schistosome worms. Earlier work by our group suggests that protective immunity against schistosomes is mainly stimulated by dying worms, and that in the short term, praziquantel treatment boosts immunity through killing worms. The longer-term impact upon the development of protective immunity is unknown. In this paper, we used a mathematical model which was able to replicate short-term patterns of infection and antibody to predict the long-term changes in antibody and infection levels that would occur during and after a 5-year treatment programme. We found that the longevity of protective immunity was particularly influential. Short-lived protective immunity was associated with levels of protective antibody declining below pre-treatment levels in the long term, and also with an increase in measured infection levels (eggs in urine) to peak above pre-treatment levels after the treatment programme finished. Antibody declines and infection peaks post-treatment were also predicted if treatment programmes reduced schistosome transmission. These results highlight the possible negative consequences of ceasing mass treatment programmes once they have commenced.

## Introduction

Urogenital schistosomiasis (caused by the blood fluke *Schistosoma haematobium*) remains a prevalent tropical disease, infecting over 100 million people in sub-Saharan Africa [1], [2]. Recent control efforts have focussed upon mass drug administration (MDA) using the antihelminthic drug praziquantel [3], [4], with the principal aim of reducing morbidity, although MDA can significantly reduce both population infection levels [5], [6] and transmission rates [5], [7]. To maintain low infection levels treatments must be repeatedly administered for an indefinite time period [8], [9].

MDA reduces infection levels directly through killing worms, and indirectly through reducing transmission. Acquired immunity enhances treatment efficacy, and influences subsequent infection dynamics [10]. Previous modelling work, which assumed protective immunity was stimulated by live worms, suggested that repeated population-level treatment would disrupt the development of acquired immunity by removing the antigenic stimulus [10], [11]; if treatment ceased, then under some circumstances, infection levels could ‘overshoot’ to exceed pre-treatment levels [10].

Protective immunity to schistosomes appears to develop slowly, with children in endemic areas experiencing repeated re-infection while adults experience much lower levels of infection, even with high exposure [12], [13]. Infection intensity peaks at an earlier age in areas with more intense transmission [14], and this is mirrored by immune responses associated with protection [15], suggesting that protective immunity is related to cumulative exposure to infection rather than age-related physiological factors. Earlier studies have shown that age-related changes in reinfection rates are explained by protective antibody levels [16], and that the development of resistance is dependent upon exposure history [17]. Several studies have demonstrated that praziquantel treatment boosts schistosome-specific antibody responses to *S. mansoni* and *S. haematobium* [18]–[20], and accelerates isotypic changes which occur more gradually with age [21], [22]. Praziquantel kills adult worms, enhancing serological recognition of *S. haematobium* antigens [23]. Increased exposure to antigens released from dying worms is thought to be responsible for stimulating these immunological changes following praziquantel treatment. Several of the responses boosted by praziquantel treatment, including IgE, IgG1, and cytokines IL-4 and IL-5, have been associated with protection against re-infection in other studies [16], [20], [24], [25], and some studies have shown that responses boosted by treatment are associated with protection against re-infection in the same population [26], [27], suggesting that treatment enhances protective immunity.

Recent mathematical modelling for *S. haematobium* showed that post-treatment boosts in antibody responses associated with protection are most consistent with protective antibody being stimulated by dying worms and reducing worm fecundity [28]. This study suggested that if protective antibody were mainly stimulated by antigens from other life stages (including cercariae, live worms, or eggs) then a boost in antibody would not be seen following treatment [28]. No models have previously looked at long-term effects of MDA upon the dynamics of protective immunity and measured egg output when such immunity is stimulated by dying (rather than live) worms. While treatment is expected to increase antigenic exposure and boost protective immune responses in the short term through worm killing, a period of reduced exposure to dying worms will follow the initial reduction in worm burden since treatment causes worms to die sooner than they naturally would. Exposure will be further reduced if population-wide treatment reduces transmission rates, decreasing re-infection. The long-term implications of mass treatment for the development of protective immunity are not fully clear [29].

Here, using a model with protective immunity stimulated by antigens released from dying *S. haematobium* worms, we assess the expected impact of MDA upon the development of acquired immunity, and upon measured egg output, both during and after a mass treatment campaign.

## Methods

### The model

We used a stochastic individual-based model which describes changes in worm burden, egg output and a protective antibody response with age for people living in an area with endemic schistosome infection. This model has been fully described previously [28]. Briefly, the model tracks the number of worms an individual harbours between their birth and 34 years of age. Individuals acquire new worms through contact with water containing infective larvae. As suggested by field studies, rates of water contact change with age [30] and vary between individuals [31].The number of cercariae acquired per water contact is independent of population infection levels and remains constant over time (unless reduced transmission is assumed during MDA). Note that transmission of parasites between humans and snail intermediate hosts is not explicitly modelled. Acquired cercariae develop into adult worms (with approximately Gaussian-distributed survival, following earlier modelling studies [32]), which produce eggs. The number of eggs within the host is assumed to be proportional to current worm burden but reduced by protective antibody. It is also assumed that egg output per worm is constant regardless of worm age. Measured egg output is calculated as the average of three ‘samples’ drawn from a negative binomial distribution around the number of within-host eggs. The protective antibody response is relatively long-lived (decay rate of 0.008–0.8 year^−1^, equivalent to a half-life of 10 months–87 years), as suggested by earlier model fitting [28]; no direct estimates are available for the longevity of protective immunity against schistosome parasites in humans, but these estimates fall between the decay rates estimated for antibody responses to other pathogens [33], and for memory B cells [34]. Protective antibody is stimulated by antigens from dying worms and reduces worm fecundity, as suggested by previous comparison of model output with field data [28], and as demonstrated for the leading schistosome vaccine candidate, a 28 kDa glutathione *S*-transferase [35]. Most of the models used here include an ‘antigen threshold’, a level of cumulative antigen exposure which must be exceeded before a protective antibody response is mounted, as suggested by previous model fitting, but we include models without an antigen threshold which have also been found to fit the data [28].

### Model parameterization and fitting

In earlier work, this model was parameterised using data from studies in Zimbabwe and elsewhere, and fitted to population data on pre- and post-treatment *S. haematobium* egg counts and specific antibody responses from several rural sites in Zimbabwe with endemic infection [28]. A grid-search of parameter space was performed to identify parameter combinations which were consistent with field data, varying the following parameters simultaneously: mean population infection rate, worm life span, antibody strength, immune response decay rate, and antigen threshold level. This grid-search was repeated, varying each of the following parameters separately: aggregation of contacts, rate of changing contact rate, aggregation of acquired cercariae, number of eggs per worm, and aggregation of egg output. Parameter combinations from all of these grid-searches which were able to reproduce cross-sectional egg output and antibody patterns and short-term post-treatment egg output and antibody dynamics were used in the current analysis to estimate the long-term impact of treatment.

### Population structure

A population of 175 individuals was simulated, with 5 individuals in each yearly age group from 1 to 34 years old at the time of the baseline survey. Individuals were simulated up to their respective ages before the initial baseline egg count and antibody levels were recorded and the first round of treatment applied. Individuals were then simulated for a further 15 years after this initial survey, during and after MDA (see next section for treatment schedules). When individuals reached the age of 34, they were replaced by 1 day old infants with no worms or antibody, to maintain a constant population size.

### Treatment schedules

Six treatment schedules were used which vary treatment frequency, target population, coverage and reduction in transmission (table 1). For the standard treatment schedule (schedule 1), treatment was given to school-aged children, defined as those aged 6–15 years old, as recommended by the WHO and implemented by the Schistosomiasis Control Initiative (SCI) [3], [4]. Treatment was applied annually for five years (five treatments in total). Annual treatment is advised for, and used in, high-prevalence communities [3], [4]. In the standard treatment schedule, coverage was assumed to be 75%, in line with WHO targets and achieved coverage in several countries [3], [36], [37], and it was assumed that treatment did not affect transmission.

**Table 1:** Treatment schedules used.

Schedule description		Treatment frequency	Target population	Treatment coverage	Effect on transmission
1	Standard	Annual	Schoolchildren (6–15 years old)	75%	None
2	Biennial	Biennial	Schoolchildren (6–15 years old)	75%	None
3	90% coverage	Annual	Schoolchildren (6–15 years old)	90%	None
4	Treat all aged 6–34 years old	Annual	Schoolchildren and adults (6–34 years old)	75%	None
5	100% transmission reduction	Annual	Schoolchildren (6–15 years old)	75%	100% reduction
6	50% transmission reduction	Annual	Schoolchildren (6–15 years old)	75%	50% reduction

In each of the other treatment schedules, one parameter was changed from the standard schedule (table 1). In schedule 2, biennial treatment (i.e. treatment every two years) was given over a five year period (three treatments in total). Biennial treatment is advised for, and used in, areas with moderate prevalence [3], [4]. Schedule 3 had 90% treatment coverage (as achieved in some countries [38], [39]). In schedule 4, the whole population over the age of 5 was treated, as recommended and implemented for high risk populations [3], [4]. In schedules 5 and 6, it was assumed that treatment reduced transmission by 100% or 50% respectively. For simplicity, treatment was assumed to reduce transmission as a step change to a fixed level, from the day after the first treatment up until one year after the final treatment, when transmission returned to its original level.

For all treatment schedules (1–6), treatment was applied over a five year period and each treatment was applied the day after egg counts and antibody levels were recorded. Treatment was applied randomly across the eligible population at the required coverage level (75% or 90%) at each round of treatment, meaning that an individual's chance of being treated in each round was independent of whether they had been treated in previous rounds. Treatment was assumed to be given independently of worm burden or egg output, in line with the usual MDA strategy of giving treatment to all school-aged children [3]. For all schedules, a treatment efficacy of 90% was assumed (90% of worms were killed), which gave reductions in egg output of 87–98%, in line with field studies [40].

### Analysis

For each parameter set, 200 repeat simulations of the whole population were run, and mean levels of egg output and antibody for the whole population aged 6–34 years old were calculated pre-treatment and at yearly intervals during and after the simulated treatment regime, averaged over the 200 repeat simulations. This age range was used in order to capture the changes in egg counts and antibody levels in treated individuals as they aged over the long follow-up period. Egg output and antibody dynamics were studied to see how quickly they returned to pre-treatment levels. The conditions (parameter values or treatment schedules) under which protective antibody levels fell below pre-treatment levels or egg counts overshot pre-treatment levels were identified.

## Results

### Standard treatment schedule (schedule 1)

#### Importance of immune decay rate and worm life span

**Figure 1:**
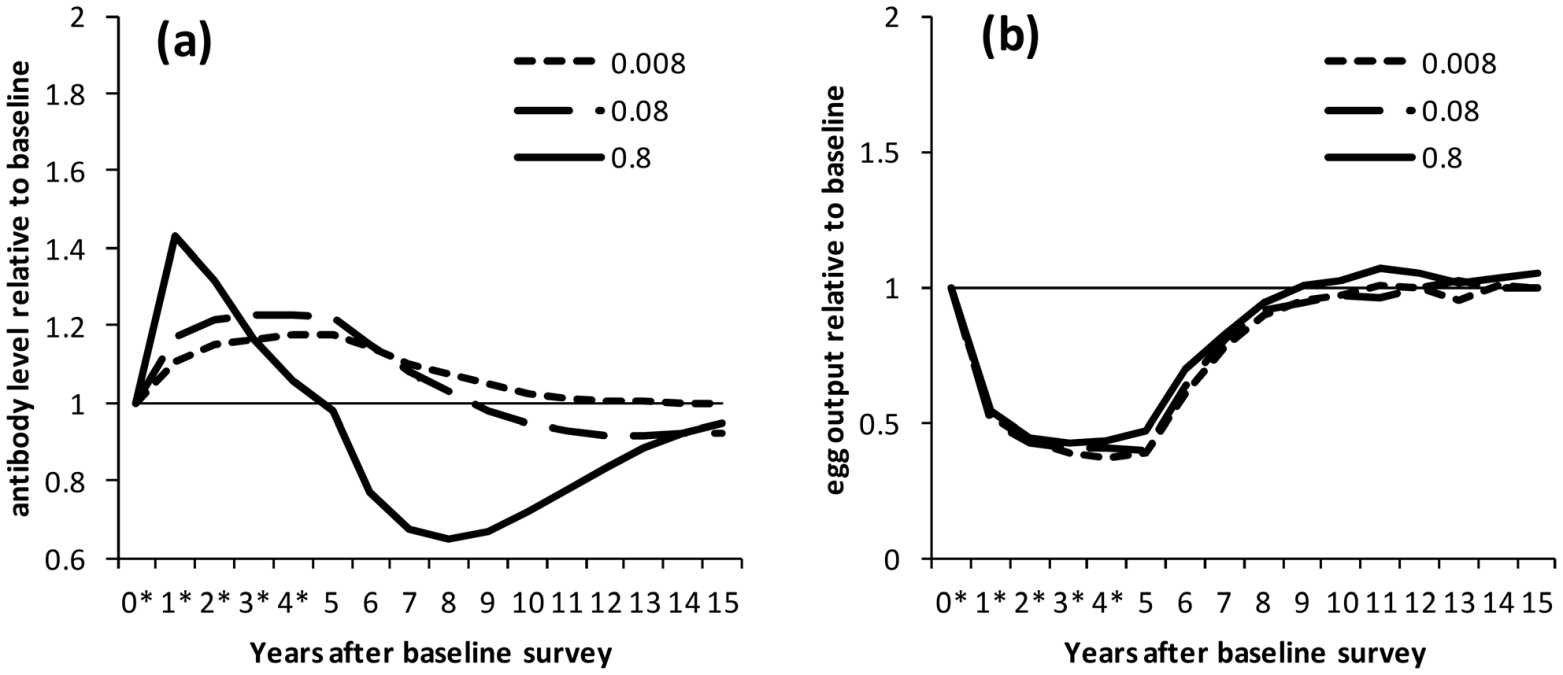
Dynamics of protective antibody and egg output during and after treatment, by immune decay rate. Results are shown for the situation where there is no reduction in transmission. Treatment was applied at yearly intervals for 5 years to school-aged children (6–15 years old) with 75% coverage. Treatment was applied the day after surveys marked *. (a) Antibody levels and (b) egg output are shown relative to pre-treatment levels for selected parameter sets which reproduced cross-sectional and post-treatment patterns in previous analyses. Results are shown separately for parameter sets with different rates of immune decay: 0.008, 0.08 and 0.8 year^−1^; for all parameter sets, worm life span is 6.5 years.

We found that protective antibody dynamics were mainly determined by immune decay rate and worm life span – this is illustrated with selected parameter sets which reflect the general patterns seen (exceptions are noted in the section on overshoots in egg output). Figure 1 shows how levels of antibody and egg output varied with immune decay rate for the same worm life span (6.5 years). Protective antibody always increased after the initial round of treatment, with the greatest relative boost, and greatest subsequent drop, seen with the most rapid immune decay rate (figure 1a; note that this figure shows *relative* changes in antibody levels. Actual antibody levels are shown for comparison in supplementary figure S1a). With rapid immune decay, antibody levels peaked one year after treatment began and declined during subsequent treatment rounds whereas models with slower immune decay saw progressive increases in antibody levels over five years of treatment (figure 1a). For most parameter sets, little difference was seen in egg output for different immune decay rates (figure 1b, figure S1b). Levels of egg output fell and then plateaued over the five rounds of treatment, and then returned to pre-treatment levels after treatment ceased, over a similar timescale for all immune decay rates (figure 1b).

**Figure 2:**
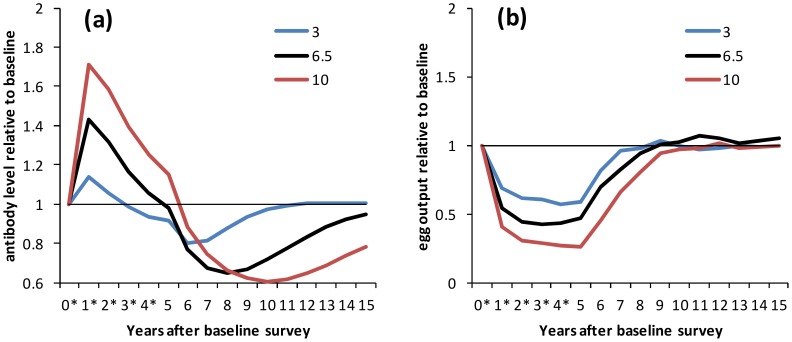
Dynamics of protective antibody and egg output during and after treatment, by worm life span. Results are shown for the situation where there is no reduction in transmission. Treatment was applied at yearly intervals for 5 years to school-aged children (6–15 years old) with 75% coverage. Treatment was applied the day after surveys marked *. (a) Antibody levels and (b) egg output are shown relative to pre-treatment levels for selected parameter sets which reproduced cross-sectional and post-treatment patterns in previous analyses. Results are shown separately for parameter sets with different mean parasite life span: 3, 6.5 and 10 years; for all parameter sets, the immune decay rate is 0.8 year^−1^.

Figure 2 shows how levels of antibody and egg output varied with parasite life span for the same level of immune decay (0.8 year^−1^). Models with a longer parasite life span showed both a higher boost and a more substantial drop in antibody levels than those with shorter parasite life span (figure 2a; similar results are seen for less rapid immune decay, figure S2a,c). Antibody levels dropped below pre-treatment levels before the fifth round of treatment if immune decay was rapid (0.8 year^−1^) and worm life span was short (3 years) (figure 2a). Egg output levels were reduced to a lesser extent, and returned to pre-treatment levels earlier, with progressively shorter worm life span (figure 2b, figure S2b,d).

#### Overshoots in egg output

**Figure 3:**
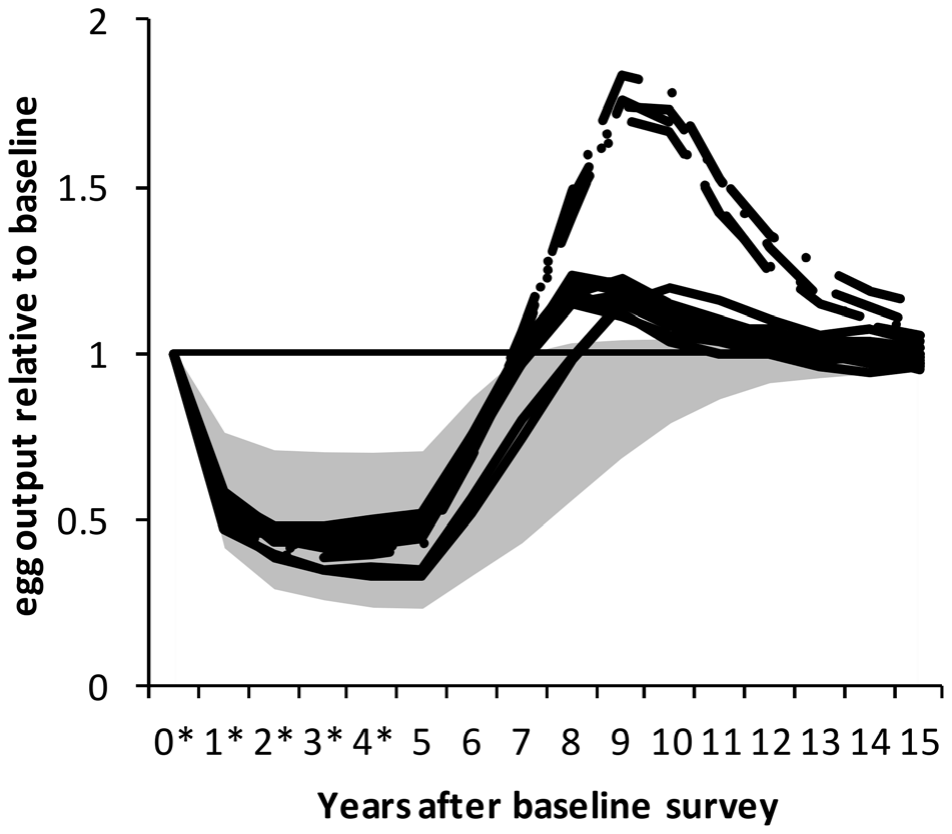
Dynamics of egg output during and after treatment when an overshoot in egg output occurs. Results are shown for the situation where there is no reduction in transmission, individually for only those parameter combinations where an overshoot in egg output levels is seen after treatment stops. Results are distinguished for models which include an antigen threshold (solid lines; n = 11) and models which do not (dot-dashed lines; n = 4). The shaded area shows the 95% credible interval for all parameter sets in which no overshoot in egg output is seen (n = 290). For all parameter sets, the immune decay rate is 0.8 year^−1^. For models including an antigen threshold, antibody strength is 0.256 per plasma cell and the antigen threshold is 25 antigen units.

For the standard treatment schedule (which assumes no reduction in transmission during MDA), some parameter combinations were identified which gave rise to overshoots in measured egg output after MDA ceased (figure 3). The overshoots were identified by eye from plots, but were found to correspond to particular parameter values. For models without an antigen threshold, all of these parameter combinations had rapid immune decay (0.8 year^−1^); for models with an antigen threshold, the parameter combinations all had rapid immune decay (0.8 year^−1^), a low antigen threshold (25 antigen units) and moderate antibody strength (0.256 units per plasma cell). Of all the unique parameter sets explored, 11/293 parameter sets for models including an antigen threshold, and 4/12 without an antigen threshold, gave rise to an infection overshoot. Models without an antigen threshold predicted higher overshoots (>70% above pre-treatment levels) than models which included an antigen threshold (where overshoots were around 14–23%; figure 3).

### Treatment schedules 2 to 4 varying treatment frequency, coverage and targeting

**Figure 4:**
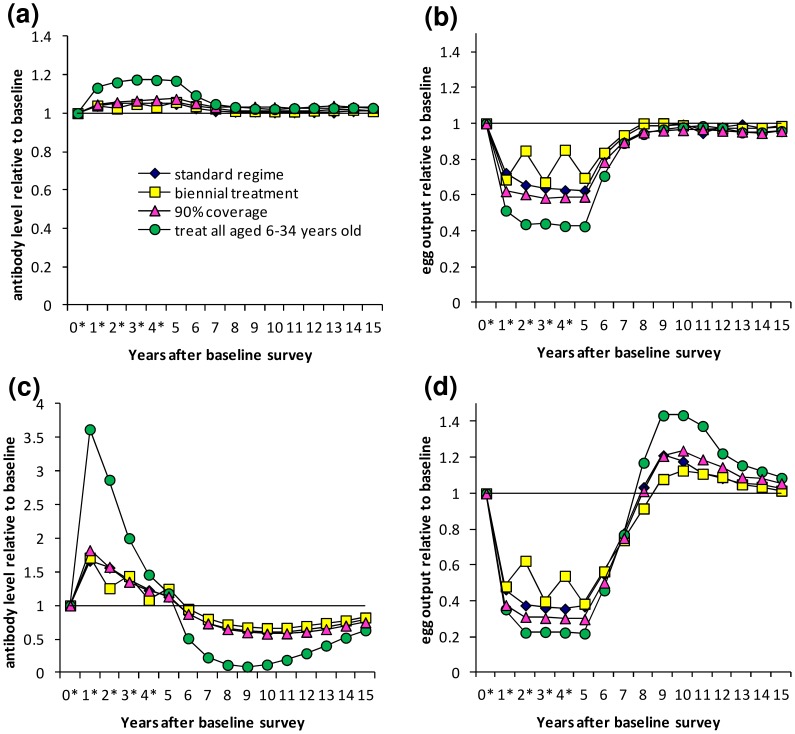
Dynamics of protective antibody and egg output under different treatment regimes. Results are shown for the situation where there is no reduction in transmission. Results are shown for (a,c) antibody and (b,d) egg output, for two different parameter combinations. Treatment was applied over a five-year period, with treatment frequency, coverage and targeting in the following combinations: blue diamonds - standard; yellow squares - biennial treatment; pink triangles - 90% coverage; green circles - treatment of 6–34 year olds. Treatment was applied the day after surveys marked * for all except biennial treatment, where treatment was applied the day after surveys 0, 2 and 4.

The impacts of separately varying the frequency of treatment and the coverage and age-range of the target population (treatment schedules 2–4) are shown for single parameter combinations (figure 4), but demonstrate trends seen for all parameter sets. Results are shown for one parameter set that did and one that did not give an overshoot in egg output for treatment schedule 1.

With biennial treatment (schedule 2), protective antibody declined and egg output increased following non-treatment years, but their levels approached those seen with annual treatment (schedule 1) one year after each treatment (figure 4). The overshoot in egg output was less pronounced for biennial versus yearly treatment (figure 4d). Changing the level of coverage of the school-aged population (90% coverage (schedule 3) vs.75% (schedule 1)) made little difference; it gave a slightly greater increase in antibody and greater reduction in egg output during treatment, and a slightly more pronounced overshoot in egg output (figure 4). Treating both adults and children (aged 6–34 years old, schedule 4) rather than just school-aged children (6–15 years old, schedule 1) gave a much larger antibody boost and greater reduction in egg output during the treatment programme and greater overshoot in egg output (figure 4).

### Treatment schedules 5 and 6 – Reduction of transmission

**Figure 5:**
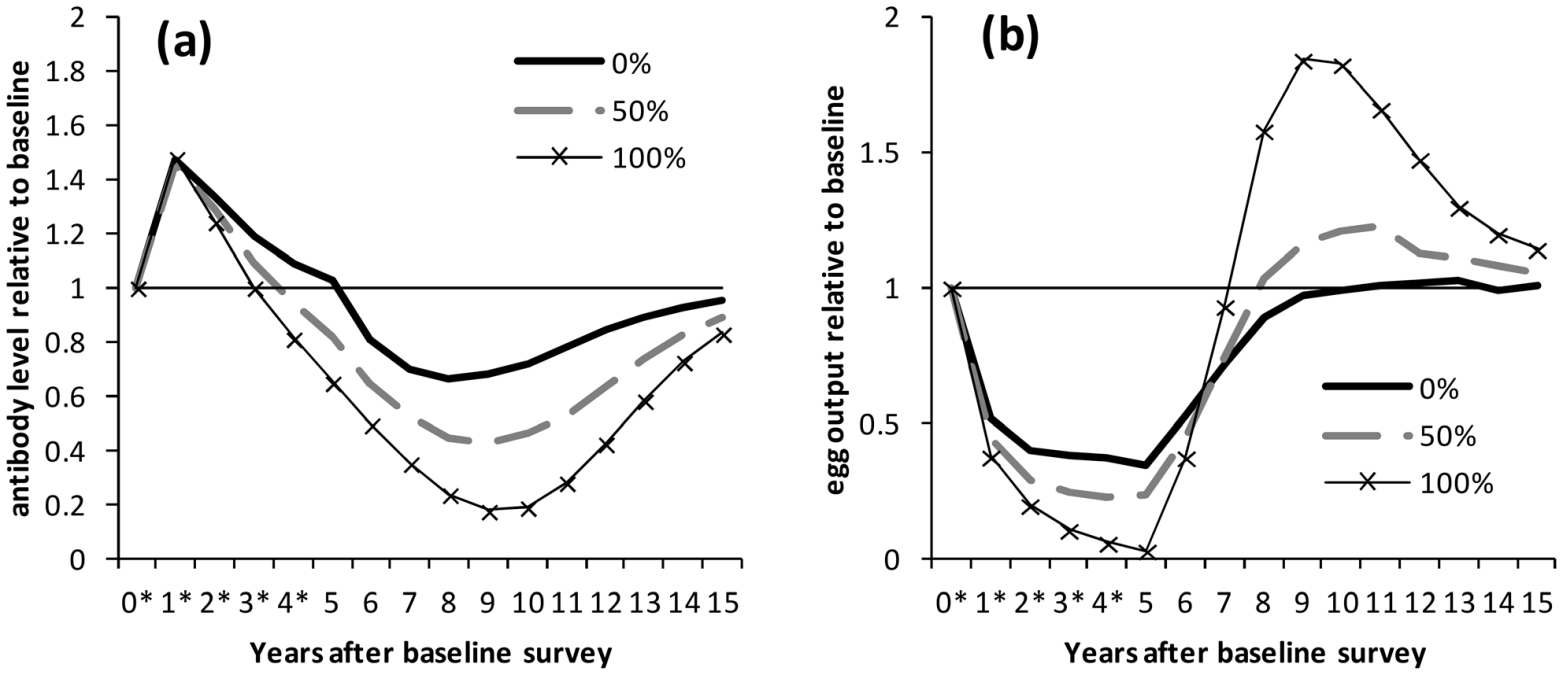
Dynamics of protective antibody and egg output during and after treatment, for different transmission assumptions. Treatment was applied at yearly intervals for 5 years to school-aged children (6–15 years old) with 75% coverage. Treatment was applied the day after surveys marked *. (a) Antibody levels and (b) egg output are shown relative to pre-treatment levels for one selected parameter set which reproduced cross-sectional and post-treatment patterns in previous analyses. Results are shown with 0, 50 or 100% transmission reduction assumed during MDA and for 1 year post-treatment. For this parameter set, the immune decay rate is 0.8 year^−1^ and worm life span is 6.5 years.

The effects of assuming that transmission is reduced during MDA are shown for one parameter set (figure 5), but these trends were seen for all of the parameter sets examined. If 100% reduction in (i.e. no) transmission was assumed during MDA (schedule 5), protective antibody always fell below pre-treatment levels at some point, before treatment ceased if there was rapid immune decay (0.8 year^−1^; figure 5a). Egg output was always reduced to below 5% of pre-treatment levels after five treatment rounds and overshoots in egg output were always predicted (figure 5b). Higher infection rates gave rise to higher and earlier overshoots in egg output (data not shown). With 50% transmission reduction (schedule 6), antibody levels always dropped below pre-treatment levels, but more slowly and to a lesser extent than when 100% reduction in transmission was assumed (figure 5a), and for most parameter sets, egg output was still predicted to overshoot pre-treatment levels, to a lesser extent than with 100% reduction in transmission (figure 5b).

## Discussion

Several MDA programs for schistosomiasis are currently underway in Africa [41]. While their immediate impact on infection and morbidity in affected individuals is unequivocal, their long-term effects on infection dynamics are not yet fully understood. Our models predict that population levels of *S. haematobium* infection will be substantially reduced by repeated MDA, while levels of protective antibody will be initially boosted by treatment, in agreement with patterns seen in the field. We predict that, in the long-term, levels of antibody could fall below pre-treatment levels after or even during MDA. More rapid declines in protective antibody levels are predicted with more rapid immune decay, shorter worm life span or reduced transmission. After the initial increased exposure to dying worms that treatment brings about, the reduced worm burden leads to a subsequent reduction in exposure to dying worms, leaving antibody levels strongly influenced by immune decay rates. Reduced transmission further reduces antigen exposure. Baseline antigenic exposure rates are expected to be lower in models with a longer worm life span, and so the reduced antigenic exposure following treatment will have a more rapid effect in models with short worm life span, leading to more rapid declines in antibody.

We found that measured egg output could rebound to levels exceeding pre-treatment levels after cessation of MDA. Our finding that this was very likely to happen if treatment temporarily reduced transmission confirms findings from earlier work using different models [10]. Importantly, we found that it could also occur in the absence of any reduction in transmission, and was more likely to occur if the immune response decayed rapidly. The fact that, without reduced transmission, a rebound in infection only happened for a restricted set of parameters, highlights how important it is to estimate these parameters to improve the accuracy of model predictions. The rate of decay of protective immunity is particularly important. Some studies (mainly on *S. mansoni*) suggest that schistosome-specific antibody levels may decline below pre-treatment levels following an initial boost, behaviour predicted for medium- or short-lived antibody responses in our model [42], [43], but this is not always seen and more accurate estimates are required.

Our results suggests that MDA might disrupt the build-up of protective immunity (or may disrupt existing immunity) against schistosomes, despite short-term boosting of this protective response. Interestingly, a reduction in antibody levels below pre-treatment levels during MDA did not necessarily correspond with overshooting of egg output after treatment ceased. It should be noted that, even when overshoots in egg output occur after treatment stops, the overall impact of the intervention on egg output (taking the overshoots into account) may still be positive; the reductions in egg output during the control programme may be sufficient to offset the increases seen after treatment stops.

We found that increasing the coverage of treatment of school children from 75% to 90% only increased population antibody levels and decreased measured infection levels by a very small amount. The random allocation of treatment at each round meant that even at 75% coverage, the chances of an individual never being treated over the five rounds of treatment were very small, which may account for the comparatively small coverage effect. Two previous modelling studies looking at *S. haematobium* in Ghana [8] and *S. japonicum* in China [44] also found little difference in long-term infection dynamics between biennial and annual treatment. However, other modelling studies have suggested different impacts of biennial versus annual treatment [9], [45]. Treating the whole population rather than just school-aged children gave a more pronounced boost to population-level protective antibody and a greater reduction in egg output during MDA, but meant that any overshooting of egg output after treatment ceased became more pronounced, suggesting that infection rebounds could be more serious following more intensive control efforts.

Previous modelling studies which considered the effects of acquired immunity on the impact of MDA suggest that the strength and duration of protective immune responses play an important role in determining infection dynamics [10], which was also found here. Our results suggest that, without any reduction in transmission post-treatment, an overshoot in measured infection levels after treatment stops is most likely to occur with relatively rapid immune decay rates (half-life of 10 months); in contrast, Chan et al. (1996) [10] reported overshoots with slow immune decay rates (half-life of 7 years), and not with more rapid decay. This discrepancy may arise because they compensated for slow immune decay rates with higher infection rates [10]. In the current analysis, when treatment was assumed to reduce transmission, higher infection rates gave rise to more pronounced overshoots in egg output.

Our results support the long-term maintenance and monitoring of existing MDA programmes, to ensure that treatment continues while transmission is still ongoing. In addition to MDA, other measures to reduce transmission should also be strengthened, including the provision of safe water and sanitation facilities, and good health education [46]–[48].

In conclusion, our models predict that, with protective immune responses stimulated by dying *S. haematobium* worms, repeated MDA will boost protective immunity initially, but antibody levels could decline below pre-treatment levels during or after MDA. In some circumstances, we also predict that post-MDA egg output could exceed pre-intervention levels. Field data are not currently available to test these predictions, but they have been made using a calibrated model which reproduces robust patterns seen in short-term pre- and post-treatment studies of *S. haematobium* infection [28]. While MDA programmes have had substantial impact upon schistosomiasis infection levels, this analysis highlights the potential negative consequences of ceasing a mass treatment programme.

## Supporting Information

Figure S1 **Dynamics of protective antibody and egg output during and after treatment, by immune decay rate: Absolute values.** The results from figure 1 are shown using absolute, rather than relative, values. (a) Antibody levels and (b) egg output are shown for selected parameter sets with different rates of immune decay: 0.008, 0.08 and 0.8 year^−1^; for all parameter sets, worm life span is 6.5 years.
(TIF)

Figure S2 **Dynamics of protective antibody and egg output during and after treatment, by worm life span, for different immune decay rates.** Similar results to figure 2 are shown for lower immune decay rates. (a,c) Antibody levels and (b,d) egg output are shown relative to pre-treatment levels for selected parameter sets which reproduced cross-sectional and post-treatment patterns in previous analyses. Results are shown separately for parameter sets with different mean parasite life span: 3, 6.5 and 10 years; the immune decay rate is (a,b) 0.08 year^−1^, (c,d) 0.008 year^−1^.
(TIF)
