# Supplementary figures and images for: Predicted Impact of Mass Drug Administration on the Development of Protective Immunity against Schistosoma haematobium
Source: PLoS Negl Trop Dis. 2014 Jul 31;8(7):e3059. doi: 10.1371/journal.pntd.0003059 (PMC4117464; doi:10.1371/journal.pntd.0003059)

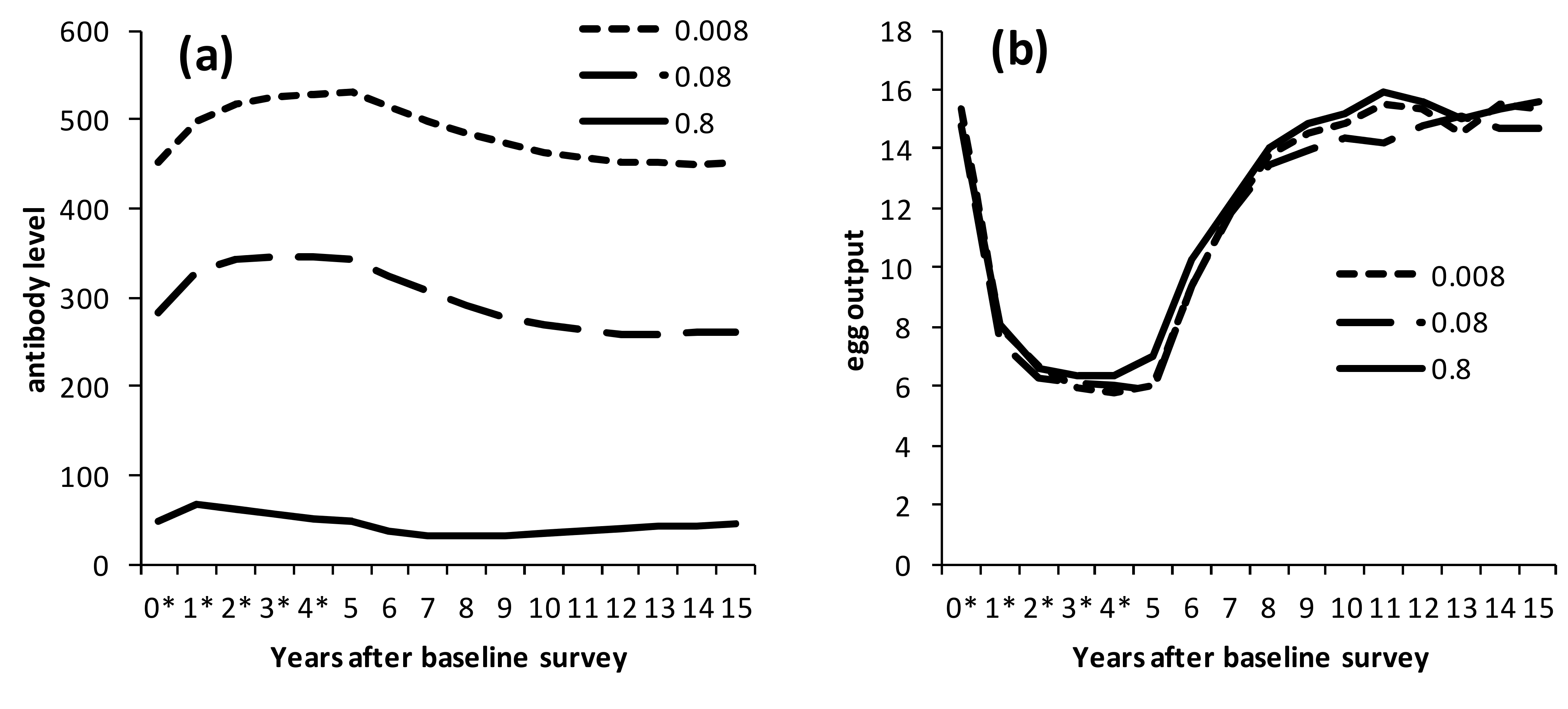

Supplement: Figure S1 — Dynamics of protective antibody and egg output during and after treatment, by immune decay rate: Absolute values. The results from figure 1 are shown using absolute, rather than relative, values. (a) Antibody levels and (b) egg output are shown for selected parameter sets with different rates of immune decay: 0.008, 0.08 and 0.8 year−1; for all parameter sets, worm life span is 6.5 years. (TIF) [file pntd.0003059.s001.tif]

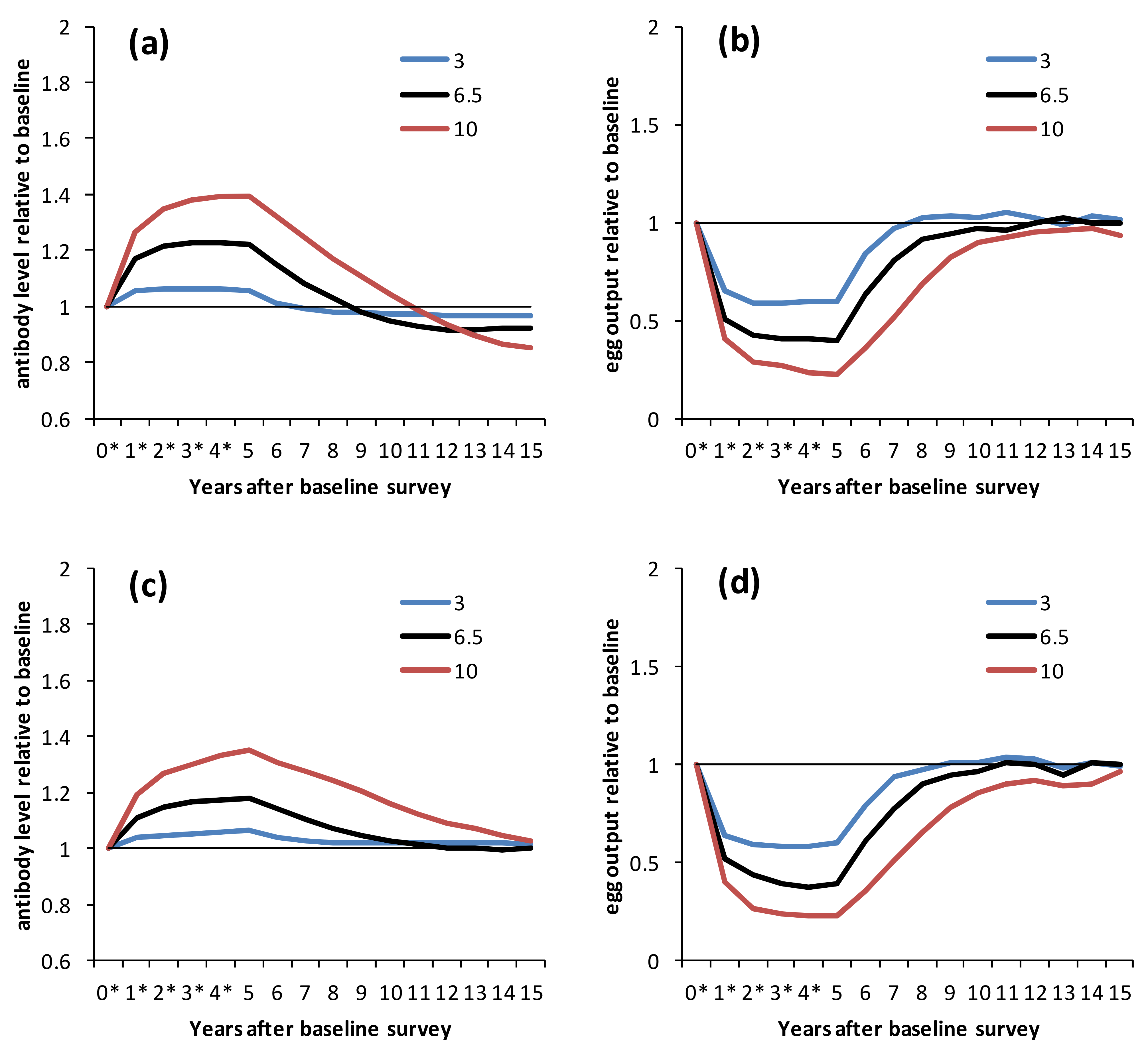

Supplement: Figure S2 — Dynamics of protective antibody and egg output during and after treatment, by worm life span, for different immune decay rates. Similar results to figure 2 are shown for lower immune decay rates. (a,c) Antibody levels and (b,d) egg output are shown relative to pre-treatment levels for selected parameter sets which reproduced cross-sectional and post-treatment patterns in previous analyses. Results are shown separately for parameter sets with different mean parasite life span: 3, 6.5 and 10 years; the immune decay rate is (a,b) 0.08 year−1, (c,d) 0.008 year−1. (TIF) [file pntd.0003059.s002.tif]
